# 3dct Conduit and Oesophageal Metrics, a Valuable Method to Diagnose Post Sleeve Gastrectomy Abnormalities

**DOI:** 10.1007/s11695-024-07528-3

**Published:** 2024-10-09

**Authors:** S. T. Alhayo, M. Guirgis, C. Siriwardene, L. Dong, S. A. Said, M. L. Talbot

**Affiliations:** 1 Upper GI Unit, Department of Surgery, St George Hospital, Kogarah, NSW 2217 Australia; 2 St George & Sutherland School of Medicine, University of New South Wales, Sydney, Australia; 3 Digestive Disease Institute, Cleveland Clinic, Cleveland, OH 44195 USA

**Keywords:** Obesity, Sleeve gastrectomy, 3DCT, Incisura angularis, Angularis stenosis, Reflux

## Abstract

**Purpose:**

Reflux after laparoscopic sleeve gastrectomy (LSG) may result from anatomical and functional anomalies in the gastric conduit. Three-dimensional CT scans (3DCT) offer a comprehensive view of gastric anatomy. This study aims to establish specific measurements associated with sleeve abnormalities to standardise the reporting of 3DCT which may help in management of LSG complications.

**Materials and Methods:**

This retrospective study analysed 64 post-LSG patients who underwent gastric 3DCT. Data included clinical demographics, pre-LSG BMI, BMI at 3DCT, and the duration between surgery and examination. Symptomatology prompts the scan and other concurrent investigations. Various 3DCT measurements were taken, including angularis angle (AA), surface area (ASA), conduit length (CL), proximal maximal surface area (PMSA), and distal maximal surface area (DMSA) of the gastric conduit. Patients were categorised based on endoscopy findings and symptomatology. Outcomes post-revisional surgery were assessed and analysed.

**Results:**

20.3% were male. Pre-LSG BMI and BMI at 3DCT were 45.57 (± 8.3) and 36.3 (± 8.7), respectively. Mean surgery-to-scan period was 6.2 years. 71.8% of patients presented with reflux, regurgitation, or dysphagia, whilst the remainder primarily exhibited weight regain. Patients with endoscopic evidence of stenosis/reflux demonstrated significantly lower gastric volume, ASA, and DMSA (*p* = 0.002 and *p* = 0.007, respectively). Oesophageal diameter above the conduit and an ASA to DMSA ratio ≤ 0.5 were negatively associated with AA (*p* = 0.008 and *p* = 0.08, respectively). Patients with improved outcomes after revisional bypass and gastrogastrostomy displayed a negative correlation with ASA and positive correlation with the ASA to PMSA ratio (≤ 0.5).

**Conclusion:**

3DCT measurements have a potential role in defining post-LSG stenosis and predicting outcomes of revisional surgery. Patients with anatomic abnormalities that are shown on CT appear to improve with anatomic correction.

**Graphical Abstract:**

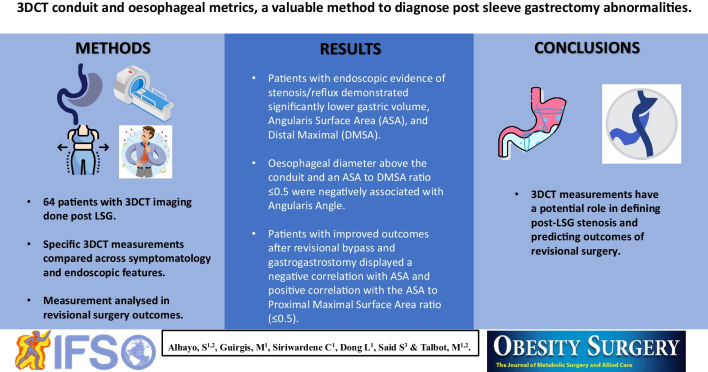

## Introduction

Laparoscopic sleeve gastrectomy (LSG) is the most popular bariatric procedure in the world [1, 2] being technically straightforward with a short operative time, recovery and good risk profile [3]. Nevertheless, it comes with complications such as bleeding, staple line leak, fistula, weight regain, strictures and stenosis [4, 5]. Later adverse outcomes such as weight regain, regurgitation and reflux may be secondary to anatomical and functional abnormalities [6].

Reflux can occur in up to 37% of sleeved patients and can be related to undiagnosed or untreated hiatus hernia, weight regain, oesophageal dysmotility and anatomical misconfiguration or combination of all [7, 8]. Anatomical malformation of the sleeve, either as a stenosis and/or poor configuration [9, 10], has been identified as a cause of ineffective food transit through the conduit [11, 12].

Stenosis is reported to occur in 0.2–4% of LSG often described as narrowing or axial obstruction at the level of angularis, which is thought to be secondary to axial rotation of the sleeved stomach, excessive angulation and/or excessive resection being performed at the time of surgery [5, 13]. Stenosis can occur within weeks or months after LSG [14].

The definition of “sleeve stenosis” has not yet been fully characterised due to failure, in part, to define what the post-LSG anatomy stomach should be. However, a number of, predominantly subjective, investigative tools are available. Endoscopy, contrast swallow studies, conventional computed tomography or magnetic resonance imaging (MRI) have all been used. Endoscopy can help in the diagnosis of reflux complications such as oesophagitis and Barrett’s, but its subjectivity and interobserver variability make it a poor method to reliably identify sleeve configuration issues. Contrast swallows whilst reasonably specific if they show failure of egress of contrast through the stomach may be insensitive [15]. MRI has been shown to be suitable however is more expensive and generally requires expert radiological interpretation when compared to other imaging. [16]. 3DCT is a low-dose radiation CT technique that does not require intravenous contrast. It has been used as a tool in determining gastric volumetry after surgery. However, other features such as hiatal hernia and sleeve configuration are more inconsistently reported because of a lack of standardisation in reporting techniques [17].

Our study explores the utility of 3DCT specific measurements of the sleeve conduit in establishing abnormalities, such as angularis stenosis and hiatus hernia in a cohort of patients undergoing investigation and management for weight regain and/or reflux after previous LSG. These measurements could be used to standardise 3DCT in the future.

## Methods

### Patients

We performed a retrospective review of electronic medical records at St George Private Hospital, Sydney, Australia, and identified 109 patients who underwent 3DCT of the stomach between January 2019 and October 2021. The 64 patients who had previously undergone laparoscopic sleeve gastrectomy and had details related to their progress and access to 3DCT imaging reported by the same radiology practice were included in the study.

Clinical and demographic data included age, gender, BMI in kg/m^2^ pre-bariatric surgery and the time between LSG and the 3DCT. All patients underwent endoscopy and other investigations undertaken as clinically indicated including barium swallow, pH and high-resolution manometry. If patients were being treated for reflux/regurgitation, their Visick scores were recorded [18].

### 3dct Scan

**Fig. 1:**
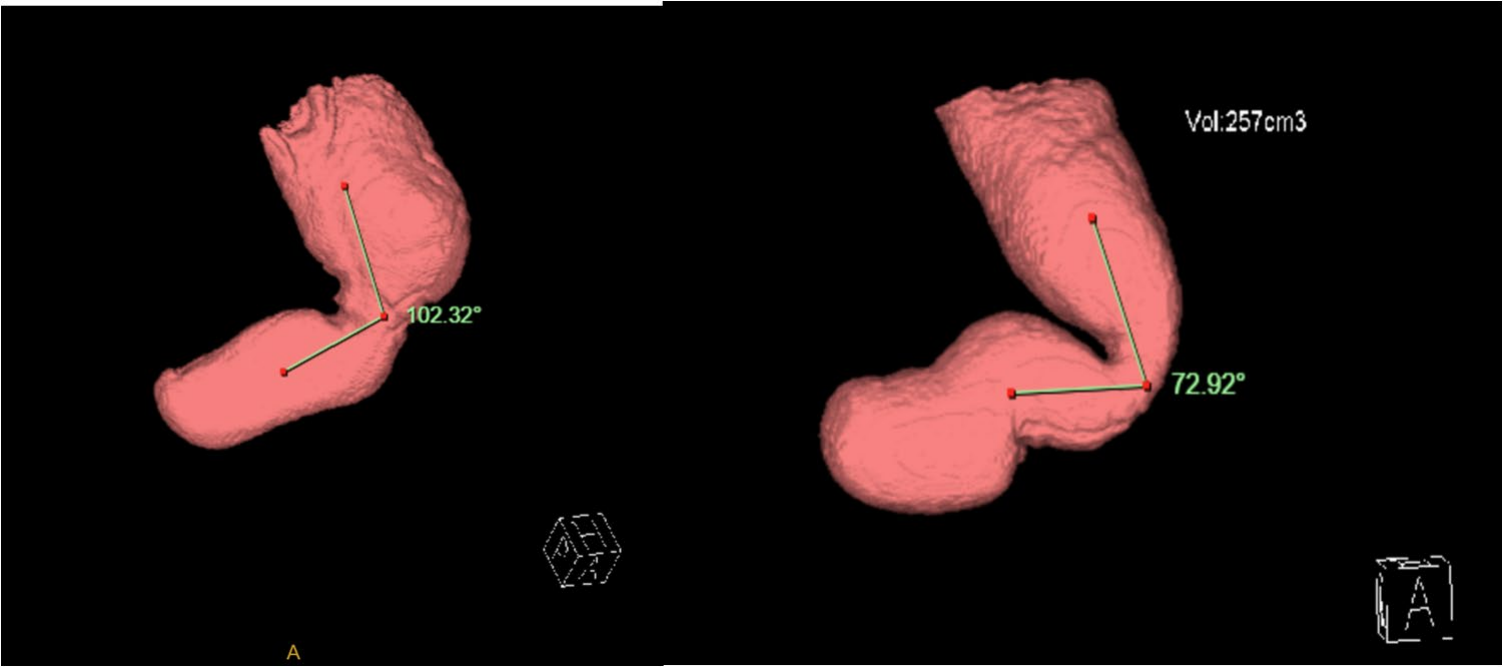
Angle measurement on 3DCT

**Fig. 2:**
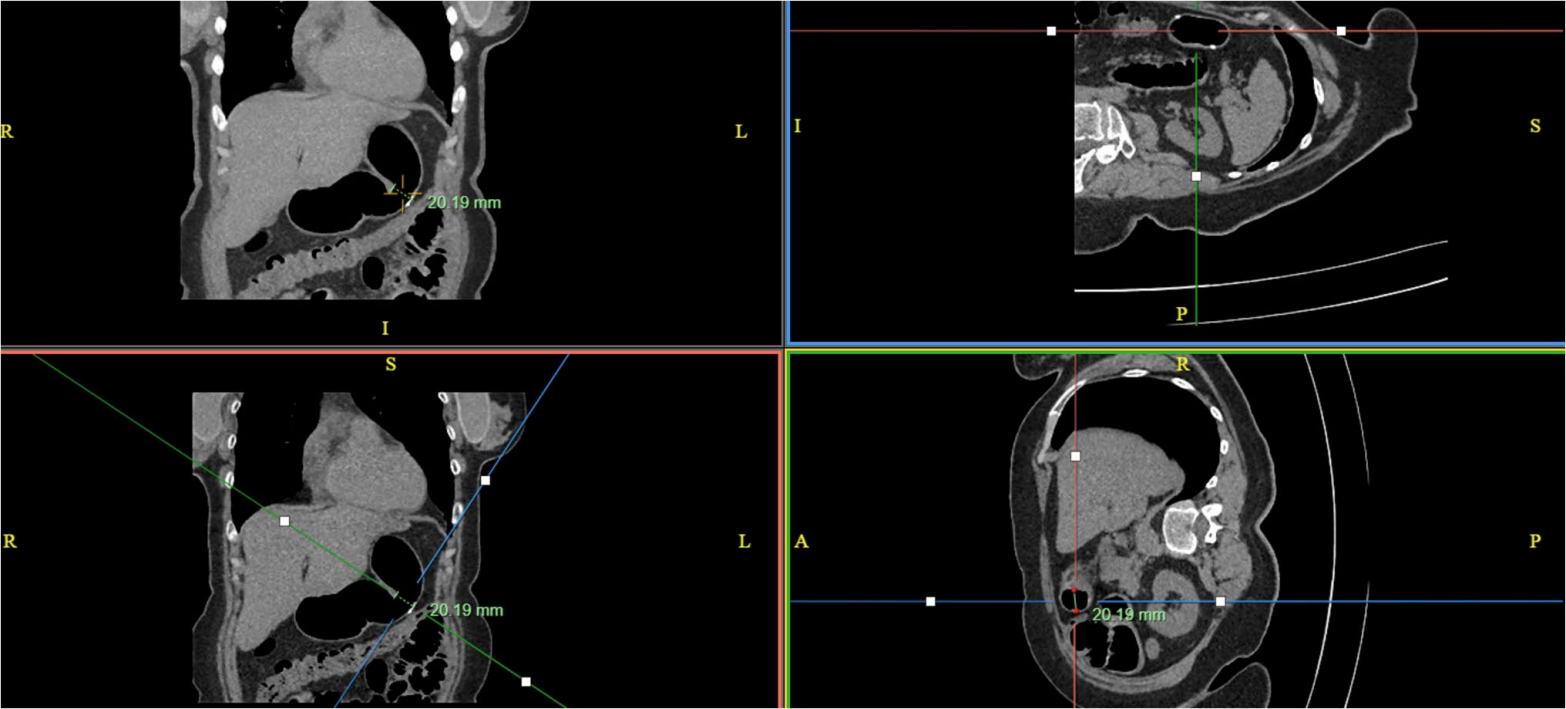
Diameter measurements on multiple 3DCT configurations

Images were reviewed using Advanced Visualisation Mode on InteleConnct Web Software. Measurements included stomach volume and length, the incisura angularis angle (IA, Fig. 1). The angle measured from the meeting of the vertical and horizontal lines of the gastric body and antrum, respectively. The incisura angularis surface area (ASA) as well as the proximal and distal maximal surface area (PMSA and DMSA, respectively) above and below the angularis were calculated from establishing the widest vertical and transverse diameters of each area. Hiatal hernia length and oesophageal diameter above the conduit were also recorded (Fig. 2). Concurrent review of images was performed to reduce interobserver variations.

### Statistical Analysis

Analysis of clinical features was performed by grouping patients according to endoscopy findings and their symptomatology that invoked further investigation using 3DCT imaging. Endoscopy results were grouped based on evidence of stenosis and/or reflux oesophagitis. 3DCT indication results were grouped to reflux/regurgitation and/or dysphagia predominant versus those with weight regain predominant. Scan parameters were analysed to assess whether there were associations between clinical presentation, endoscopic findings and gastric morphology, for example, whether relative narrowing of the angularis (as expressed by a ratio of ASA and PMSA) is associated with reflux symptoms.

Continuous variables are represented as mean and standard deviation (*x̄* ± SD) or median and interquartile range (*M*[Q1–Q3]) as appropriate. Categorical variables are presented as frequency and percentages. Univariate analysis for categorical variables was tested with chi-square or Fisher exact test, as appropriate. Normality of distribution and homogeneity of variance were tested with Shapiro–Wilk and Levene’s tests, respectively, and accordingly parametric or nonparametric methods with the appropriate variance assumption were used.

For the multivariable analysis, the 3DCT scan variables were fitted into a logistical regression model and adjusted for age and gender to test association with endoscopic stenosis or stricture. Collinearity was assessed by the variance inflation factor method. Variable forward and backward selection based on the AIC method was used. The final model was used to predict the endoscopic group, and the model concordance was estimated using the receiver operative characteristic (ROC) curve method.

For this type of study, formal consent is not required and the study protocol was approved by the Human Research and Ethics Committee at Ramsay Health, Reference no. 2012–012 (SA02928).

## Results

### Demographics and Clinical Outcomes

Of the 64 patients included in the study (Table 1), 51 were females (79.6%). Surgery to scan period was 6.2 ± 6.9 years and the average age was 46.1 ± 10.7. Preoperative BMI was 45.6 ± 8.3 and BMI at 3DCT scan was 36.3 ± 8.7. Reflux was present in 26 (40%) patients prior to their LSG and 14 (53%) of these patients were using regular proton pump inhibitors. Sixteen (25%) patients had hiatus hernia repair and another two had removal of gastric band at the time of their LSG. Two patients had experienced staple-line leak following their LSG procedure.

**Table 1:** Patient demographics and characteristics

Variable		Patient characteristics (64)
Age (years) (mean + − SD)		46.16 ± 10.71
Male (*n*, %)		13 (21.4%)
Surgery to scan (years) (mean + − SD)		6.20 ± 6.96
Preoperative BMI (kg/m^2^) (mean + / − SD)		45.57 ± 8.29
BMI at 3DCT (kg/m^2^) (mean + / − SD)		36.33 ± 8.76
Preoperative reflux (*n*, %)	Total	26 (40.6%)
	On regular PPIs (*n*, %)	14 (21.8%)
Complications (*n*, %)		2 (3%)
Type of procedure (*n*, %)	LSG alone	46 (43.7%)
	LSG + hiatus hernia repair	16 (25%)
	LSG + removal of gastric band	2 (3%)
Post LSG-revisional surgery (*n*, %)	Hiatus hernia repair	9 (33.3%)
	Bypass, gastrogastrostomy, re-sleeve	18 (66.6%)

Post-LSG, 46 (72%) patients presented with symptoms of reflux, regurgitation and/or dysphagia, with only 9 patients identified as having stenosis and/or reflux oesophagitis on endoscopy. On the other hand, endoscopic findings of reflux/stenosis correlated highly with symptoms of reflux, regurgitation and/or dysphagia (*p* = 0.042). Patients presenting primarily with dysphagia/reflux symptoms tended to have a lower BMI, concordance index of 0.658. The remaining patients presented with weight regain as their dominant concern. Table 5 describes analysis of scan findings per presentation. Furthermore, hiatal hernia was present in 39% of patients with weight regain as the primary indication for 3DCT and 59% of those with reflux/regurgitation (Table 5).

Subsequent to their 3DCT, 27 patients (42.2%) underwent revisional surgery. The majority (21 patients) underwent surgery for reflux, regurgitation and/or dysphagia symptoms, whereas only 6 patients underwent surgery for weight regain as the primary indication. Nine patients with reflux and a normal sleeve conduit without a requirement for further weight loss underwent hiatus hernia repair, four patients had gastrogastrostomy as a stricturoplasty procedure for an angularis stenosis, one a re-sleeve (1) for anatomical correction and the remaining 13 had gastric bypass (combined with hiatal repair in 2 patients). After revisional surgery, 16 (59.2%) of the patients were Visick 1, 6 (22.2%) had Visick 2, 4 (14.8%) had Visick 3 and 1 (3.7%) had Visick 4 symptom scores.

### 3dct Findings

On analysing the 3DCT characteristics (Table 2), it was found that angularis angle correlated with angularis surface area (*p* = 0.001), ASA/DMSA ratio of ≤ 0.5 (*p* = 0.08), so that patients with a more acute angle were more likely to have a narrow angularis and a narrow distal stomach. In addition, acute angulation was also found to be associated with a dilated oesophagus above the conduit (*p* = 0.008) and also with a longer sleeve.

**Table 2:** 3DCT characteristics and test between angle and other measurements. Values in bold indicate statistically significant finding

Measurements	Measurements (mean ± SD)	*Coefficient (95% CI)*	*p* value
Volumetry (ml^3^)	270.89 ± 124.06	0.954 (− 0.379–2.287)	0.547
Angularis surface area (cm^2^)	**17.49 ± 9.46**	**0.208 (0.81**–0**.335)**	**0.003**
Maximum proximal surface area (cm^2^)	35.52 ± 15.54	0.058 (− 0.181–0.296)	0.37
Maximum distal surface area (cm^2^)	26.44 ± 11.31	0.04 (− 0.113–0.194)	0.899
Length of conduit (cm)	26.7 ± 4.02	–	0.786
Length of conduit including hiatal length (cm)	**27.9 ± 5.4**	− **0.063 (**− **0.179**–**0.054)**	**0.01**
Hiatus hernia (*n*)	34	− 0.004 (− 0.011–0.004)	0.327
Oesophageal diameter (mm)	**20.1 ± 6.2**	− **0.110 (**− **0.198**–**0.022)**	**0.008**
ASA/PMSA (≤ 0.5) (*n*)	34	− 6.13 (− 15.23–2.97)	0.183
ASA/DMSA (≤ 0.5) (*n*)	**21**	− **12.8 (**− **21.13**–**4.47)**	**0.08**
Angularis angle	71.85 ± 18.29	–	–

Distal maximum surface area had strong positive correlation with volumetry (*p* = 0.005) and a weak but positive correlation with conduit length (*p* = 0.057). In addition, there was strong positive correlation between volumetry and length of conduit (*p* = 0.004). Time since surgery also correlated with conduit volume (*p* = 0.08) and proximal maximum surface area (*p* = 0.22) (and length), suggesting that the post-LSG stomach may lengthen and proximally dilate over time.

In our study, correlation existed between 3DCT and endoscopy. All patients underwent endoscopic examination. Nine of whom were identified as having either an angularis stenosis (5) and/or reflux oesophagitis (3); the remaining had either hiatus hernia only or normal endoscopy. Furthermore, endoscopy diagnosed hiatal hernia in 22 (34%) patients. 3DCT revised the diagnosis up to 31 (48%) patients. Therefore, endoscopy was specific for hiatus hernia but not as sensitive as CT. Patients with longer duration between surgery and 3D scan were more likely to have reflux/stenosis on endoscopy (*p* = 0.02). Reflux symptoms and proton pump inhibitor (PPI) use were equally present amongst patients with and without hiatus hernia whether diagnosed on endoscopy or 3DCT (Table 3), suggesting that multiple potential drivers for reflux can exist in these patients.

**Table 3:** 3DCT and endoscopy finding of hiatus hernia with reflux symptom distribution. Values in bold indicate statistically significant finding

Investigation modality	Symptoms and medications	Hiatus hernia	No hiatus hernia	Sig
3DCT		34	30	
Reflux		27	20	0.802
+ PPI		7	10	0.773
Endoscopy		22	42	
Reflux		17	29	0.532
+ PPI		5	13	0.363

Gastric volume was higher in those with normal endoscopy (*p* = 0.009). Both angularis surface area and maximum distal surface area were significantly smaller in those with endoscopic stenosis (*p* = 0.002 and *p* = 0.007), respectively. In addition, we found an association of ASA/PMSA ratio of ≤ 0.5 to endoscopic evidence of stenosis/reflux, where 8 out of 9 patients had that ratio, as opposed to 26/44 of patients with normal or HH only finding on endoscopy (*p* = 0.02) (Table 4). 5/46 (8%) patients with reflux had hernia 0–2 cm length HH and another 15 (20%) had HH that was 2–4 cm in length whilst 6 (9%) had HH > 4 cm. Of the patients with endoscopic finding of stenosis/oesophagitis, there were 5 patients with concomitant HH.

**Table 4:** 3DCT scan and patient characteristics per endoscopy findings. Values in bold indicate statistically significant finding

Variables	All (64)	Stenosis/reflux (9)	Normal ± HH (55)	*OR*	*p* value
Age (yrs)	46.1 ± 10.7	43.3 ± 12.1	46.6 ± 10.5	1.049	0.398
Male gender (*n*, %)	13 (21.4%)	0 (0%)	13 (100%)	0.577	0.102
Preoperative BMI (kg/m^2^)	45.5 ± 8.2	46.9 ± 8.6	45.3 ± 8.3	0.966	0.392
BMI at 3DCT (kg/m^2^)	36.3 ± 8.7	33.46 ± 7.62	36.78 ± 8.91	0.990	0.341
Volumetry (ml^3^)	**270.8 ± 124.0**	**179.6 ± 52.9**	**286.1 ± 126.2**	**1.012**	**0.009**
Angle (degrees)	71.85 ± 18.2	66.33 ± 13.2	72.7 ± 18.9	1.031	0.288
Angularis surface area (cm^2^)	**17.4 ± 9.4**	**10.12 ± 4.24**	**18.7 ± 9.5**	**1.287**	**0.002**
Maximum proximal surface area (cm^2^)	35.5 ± 15.5	33.5 ± 15.4	35.8 ± 15.6	0.988	0.482
Maximum distal surface area (cm^2^)	**26.4 ± 11.3**	**21.2 ± 4.0**	**27.3 ± 11.9**	**1.073**	**0.007**
ASA/PMSA ≤ 0.5	**34**	**8**	**26**		**0.02**
Conduit length (cm)	26.7 ± 40.2	26.9 ± 39.5	25.4 ± 14.7	0.956	0.313
Hiatus hernia length (cm)				1.049	
Hiatus hernia volume (ml^3^)				0.952	
Conduit length including hiatus (cm)	27.9 ± 5.4	26.9 ± 53.2	28.1 ± 5.5	0.942	.535
Oesophageal diameter (mm)	20.1 ± 6.2	20.8 ± 6.7	20.0 ± 6.2	0.970	.73
Hiatus hernia *n* (%)	34 (53.1%)	6 (4.7%)	28 (29.2%)	0.496	0.378
Surgery to scan period (yrs)	**6.2 ± 6.9**	**8.8 ± 4.1**	**6.0 ± 7.7**	**1.022**	**0.029**

Of note, 10 (55%) patients of those presented weight regain only as opposed to 24 (50%) of patients with reflux/regurgitation and/or dysphagia patients had an ASA/PMSA ratio < 0.5. Those with reflux symptoms also had slightly smaller angularis surface area and longer conduit but none reached statistical significance (Table 5).

**Table 5:** 3DCT scan findings and patient characteristics per presenting symptoms. Values in bold indicate statistically significant finding

Variables	Weight regain only (18)	Reflux, regurg ± dysphagia (46)	*OR*	*p* value
Age (yrs)	45.3 ± 10.7	46.4 ± 10.8	1.026	0.704
Male gender (*n*, %)	5 (38.4%)	8 (61.5%)	0.467	0.353
Preoperative BMI (kg/m^2^)	46.8 ± 6.9	45.0 ± 8.8	1.056	0.251
BMI at 3DCT (kg/m^2^)	**40.3 ± 10.4**	**34.5 ± 7.4**	**0.878**	**0.074**
Volumetry (ml^3^)	297.0 ± 110.1	260.4 ± 128.8	0.992	0.138
Angle (degrees)	72.5 ± 17.4	71.6 ± 18.8	1.008	0.782
Angularis surface area (cm^2^)	19.1 ± 9.0	16.8 ± 9.6	1.002	0.232
Maximum proximal surface area (cm^2^)	**40.6 ± 16.2**	**33.6 ± 15.0**	**0.973**	**0.093**
Maximum distal surface area (cm^2^)	26.2 ± 12.6	26.5 ± 10.9	0.972	0.93
Conduit length (cm)	25.6 ± 3.4	27.1 ± 4.2	1.122	0.204
Conduit length including hiatus (cm)	26.8 ± 3.6	28.3 ± 5.9	1.122	0.35
ASA/PMSA ≤ 0.5	10	24	1.15	0.807
Oesophageal diameter (mm)	19.6 ± 8.5	20.3 ± 5.2	1.019	0.659
Hiatus hernia (*n*, %)	7 (39%)	27 (59%)	1.993	0.154
Surgery to scan period (yrs)	7.05 ± 2.7	6.0 ± 3.9	0.993	0.336

### Revisional Surgery

This group of patients had lower angularis surface area than the general cohort (14.6 vs. 19.8 cm^2^, *p* = 0.02). 20/46 (43%) of patients presenting with reflux underwent revision surgery and 9/18 (50%) of patients presenting mainly for weight regain underwent revision surgery. In those who underwent revision for reflux, only 9 (33%) patients underwent hiatus hernia repair, whilst the rest had gastric bypass surgery or stricturoplasty. When grouping patients according to their outcomes, we found that patients who underwent revisional gastric bypass, gastrogastrostomy and re-do sleeve, with post-procedure Visick 1 and 2, had greater oesophageal diameter, angularis angle, but smaller proximal and distal max surface area, angularis surface area and lower ASA/PMSA ratio, than those with Visick 3 and 4. Furthermore, more patients had ASA/PMSA ≤ 0.5 who had a Visick score 1 and 2, than those who had Visick score 3 and 4. The numbers however were not enough to establish a reliable statistical significance (Table 6). This suggests that the bypass and anatomical corrective revisional surgery had maximum effect in patients with 3DCT evidence of disproportionate gastric morphology.

**Table 6:** 3DCT scan findings and symptom improvement post-LSG revisional surgery for reflux

3DCT measurements	Hiatus hernia repair only (9)		Gastric bypass and others (18)	
	Visick 1 and 2 (6)	Visick 3 and 4 (3)	Visick 1 and 2 (16)	Visick 3 and 4 (2)
Oesophageal diameter (mm)	17.4	18.4	21.9	17.5
Angularis angle	76.4	72.3	70	64.5
Angularis surface area (cm^2^)	13.3	12.9	12.9	16.4
Proximal max surface area (cm^2^)	26.3	29.9	36.7	49.4
Distal max surface area (cm^2^)	21.1	30.6	26.2	30.3
ASA/PMSA ≤ 0.5	3	2	10	1
ASA/DMSA ≤ 0.5	3	2	7	1

## Discussion

Reflux is a bothersome outcome of LSG, and its aetiology is still not well understood. De novo reflux or worsening reflux is reported to be between 8 and 50% of all post sleeve gastrectomy patients [8, 19, 20].

Reflux can be related to defective lower oesophageal sphincter anatomy and function because of hiatus hernia, whilst some studies show good outcomes of hiatus hernia repair following LSG [20, 21]. Others suggest that reflux is not improved unless the hiatus hernia repaired is larger than 4 cm [12, 19, 21, 22]. In our study, reflux symptoms were present in both those who had hiatus hernia and those who did not. Further sub-analysis showed no correlation between symptomatology and hiatus hernia size; however, 6 (66.6%) of the patients undergoing hiatal repair alone reported a post-op Visick score of less than 3.

Weight regain is another risk factor for reflux; however, there was concordance for lower BMI at representation in patients presenting with reflux, regurgitation/dysphagia than those with weight regain in our study (*p* = 0.07). Other studies have also found that reflux was also independent of weight regain in LSG patients [23, 24].

There are increasing arguments that sleeve shape impacts the function of the sleeve and therefore might present a combined anatomical and functional cause of concomitant reflux [20, 25, 26]. Laplace’s law explains that the pressure inside an inflated and elastic container with a curved surface, e.g. a bubble or a blood vessel, is inversely proportional to the radius as long as the surface tension is stable, whilst Poiseuille’s law indicates that the flow rate is directly proportional to the pressure difference and to the fourth power of the radius of the pipe or vessel. This means that even small changes in the radius can have a significant impact on the flow rate [27] and that a dilated pipe, proximal to a stenosis, will be prone to more dilation.

Anatomic abnormalities in the LSG can be in the form of an internal indentation and/or a sharp angulation of the gastric lumen, which creates a flap valve producing a functional obstruction, typically at the incisura, hence angularis incisura stenosis [13, 24]. The narrowing or stenosis could be the result of over-tight stapling and/or twisting of the sleeve from misalignment of staple lines [19, 28]. An alternate or contributing cause could arise in the years following the procedure whereby the vertical part of the sleeve dilates and the sleeve tube lengthens, thereby creating a more acute angularis angle and relative stenosis. This will lead to increased proximal gastric pressures based on the above-mentioned laws [29]. The most prevalent surgical treatment for post-LSG reflux is conversion to gastric bypass which promotes improvement in reflux control via the putative mechanisms of reduced parietal cell mass, rapid transit time through to stomach and reduced intragastric pressures [22, 23]. Whilst proving the anatomic causes of reflux in LSG can be challenging [5, 14], very few surgeons would be happy to construct a gastro-enterostomy anastomosis below a gastric stenosis if their aim was to improve reflux symptoms.

Endoscopic evaluation is a valuable tool in the diagnosis of abnormalities such as reflux it can prove to be less sensitive in establishing narrowing of conduit, where passage of the scope could be interpreted as a normal examination. This comes in addition to the potential surgeons’ bias playing a role in under-interpreting a technical error which may contribute to patients’ symptoms [15, 30].

Our study identified 2 (3.1%) endoscopic evidence of stenosis, which is similar to reported incidence of up to 3.9% in other studies. However, there were other 7 (11.9%) patients in our cohort with endoscopic evidence of reflux without presence of other contributing factors on the endoscopy. 3DCT however was able, in these patients, to suggest a potential anatomic cause.

3D computed tomography scans can help determine gastric volume; the presence of hiatus hernia with the highest accuracy [31] can also provide detailed information of the shape and measurements of the sleeve [31, 32] including detection of relative or functional stenoses which may be missed on endoscopy or other studies.

If we follow the rules of physics of angulated non-reinforced tubes, we can deduct that the creation of a narrow distal sleeve might impact on its shape and increase the angulation at the incisura, therefore reducing the surface area at that point and creating a significant pressure gradient [33]. A higher proximal pressure may lead to subsequent proximal stretch and lengthening of the conduit overtime, similar to what is seen in vascular biomechanics [34]. The results of our study indicate a reduction of angularis surface area when sharper angulation of the sleeve occurs. Furthermore, the sharper the angle the wider the oesophageal diameter above the hiatus was (*p* = 0*.*008) and the longer the conduit length (*p* = 0.01). We also see a significant positive correlation between the sleeve lengthening and duration of primary surgery to 3DCT (*p* = 0.024). Similarly, patients with endoscopic evidence of stenosis and/or reflux presented long after those with normal or hiatus hernia only. All of this corresponds to previously reported timing of presentation of patients with stenosis that can take months or years to occur [5, 14, 35].

When we compare 3DCT findings to endoscopic findings, we find lower volumetry, angularis and distal surface area in those diagnosed with stenosis and/or reflux on endoscopy (Table 5). In specific, we found more patients with disparity in the shape of the sleeve in the form of (ASA/PMSA ≤ 0.5) in those diagnosed with stenosis and/or reflux on endoscopy (*p* = 0*.*02).

In those who underwent sleeve to bypass/or stricturoplasty for reflux, patients experienced better results if they had a disparity between angularis surface area and proximal conduit surface area, in particular more patients experienced better outcome if they had an ASA/PMSA ratio of 0.5 or less. Whilst bypass in general is thought to have a better therapeutic profile for management of sleeve reflux [36], our findings suggest that patients with a detectible stenosis did well if the surgery addressed the stenosis, whereas patients without an anatomic abnormality did less well.

Our study is limited by a retrospective design, a relatively small cohort of patients and lack of control of 3DCT imaging in asymptomatic patients post sleeve. Performing a prospective study with inclusion of asymptomatic patients would further help standardise measurements of the conduit and reduce bias. In addition, adding manometry might help establish proximal gastric pressures in patients with possible gastric obstruction and therefore would add another confirmatory test to compare with the CT findings.

## Conclusion

Reflux in post LSG is multifactorial, and whilst presence of hiatus hernia can be important in some patients, other anatomic features can be a driver of pro-reflux physiologies. Natural history studies showing how the post-LSG stomach may change over time are lacking, as are studies objectively describing the relationships between reflux symptoms, anatomy, oesophageal function, intra-gastric pressures, and oesophageal acid exposure.

3DCT can identify gastric sleeve anatomy beyond gastric volume to make it a “one stop” objective tool. Hiatus hernia and its length, oesophageal diameter, angularis angle and its cross-sectional area relative to stomach above it as well as any disparities between diameters between the transverse and vertical sleeve components can all be easily calculated and reported when assessing a patient presenting with post-LSG reflux. Therefore, reporting them could help standardise post-LSG surgery assessment which may play a role in subsequent patient care.

Our study demonstrates interaction between mentioned parameters and relationship with endoscopic evidence of stenosis and/or reflux oesophagitis. Furthermore, disparity of cross-sectional areas and oesophageal diameter can play a role in predicting outcomes following revisional bariatric surgery.

A future prospective study inclusive of larger cohort of symptomatic and asymptomatic patients could help validate the clinical implications of these 3DCT parameters .
